# Alcohol consumption patterns in Thailand and their relationship with non-communicable disease

**DOI:** 10.1186/s12889-015-2662-9

**Published:** 2015-12-24

**Authors:** Mami Wakabayashi, Rebecca McKetin, Cathy Banwell, Vasoontara Yiengprugsawan, Matthew Kelly, Sam-ang Seubsman, Hiroyasu Iso, Adrian Sleigh, Thai Cohort Study Team

**Affiliations:** Public Health, Department of Social and Environmental Medicine, Graduate School of Medicine, Osaka University, 2-2 Yamadaoka, Suita-shi Osaka, 565-0871 Japan; National Centre for Epidemiology and Population Health, Research School of Population Health, Australian National University, Canberra, Australia; Centre for Research on Ageing, Health and Wellbeing, Research School of Population Health, Australian National University, Canberra, Australia; School of Human Ecology, The Sukhothai Thammathirat Open University, Bankgok, Nonthaburi Thailand

**Keywords:** Alcohol consumption, Non-communicable diseases, Thailand, Chronic disease, Hypertension, Hypercholesterolemia, Liver disease, Obesity, Binge drinking, Cross-sectional studies

## Abstract

**Background:**

Heavy alcohol consumption is an established risk factor for non-communicable diseases (NCDs) but few studies have investigated drinking and disease risk in middle income, non-western countries. We report on the relationship between alcohol consumption and NCDs in Thailand.

**Methods:**

A nationwide cross sectional survey was conducted of 87,151 Thai adult open university students aged 15 to 87 years (mean age 30.5 years) who were recruited into the Thai Cohort Study. Participants were categorized as never having drunk alcohol (*n* = 22,527), as being occasional drinkers who drank infrequently but heavily (4+ glasses/occasion - occasional heavy drinkers, *n* = 24,152) or drank infrequently and less heavily (<4 glasses/occasion - occasional light drinkers, *n* = 26,861). Current regular drinkers were subdivided into those who either drank heavily (4 + glasses per occasion - regular heavy drinkers, *n* = 3,675) or those who drank less (<4 glasses/occasion -regular light drinkers, *n* = 490). There were 7,548 ex-drinkers in the study. Outcomes were lifetime diagnoses of self-reported NCDs and obesity (body mass index ≥ 25).

**Results:**

Most women were never drinkers (40 % among females) or occasional light drinkers (39 %), in contrast to men (11 % and 22 %, respectively). Alcohol consumption was associated with urban in-migration and other recognized risks for NCDs (sedentary lifestyle and poor diet). After adjustment for these factors the odds ratios (ORs) for several NCDs outcomes - high cholesterol, hypertension, and liver disease - were significantly elevated among both occasional heavy drinkers (1.2 to 1.5) and regular heavy drinkers (1.5 to 2.0) relative to never drinkers.

**Conclusions:**

Heavy alcohol consumption of 4 or more glasses per occasion, even if the occasions were infrequent, was associated with elevated risk of NCDs in Thailand. These results highlight the need for strategies in Thailand to reduce the quantity of alcohol consumed to prevent alcohol-related disease. Thailand is fortunate that most of the female population is culturally protected from drinking and this national public good should be endorsed and supported.

## Background

Alcohol consumption globally accounted for an estimated 2.7 million deaths and 3.9 % of Disability Adjusted Life Years (DALYs) in 2010 [1]. It had a causal impact on the burden of non-communicable disease (NCDs), including several types of cancer, hypertension, ischemic heart disease, ischemic and haemorrhagic stroke and liver cirrhosis [2, 3]. Recently, attention has turned toward understanding the impact of specific drinking patterns on these health outcomes [4]. For example, while light to moderate alcohol consumption may protect from ischemic heart disease [4, 5], heavy episodic drinking (also known as “binge drinking”, or drinking in excess of 4–5 glasses on one occasion [6]) increases the risk of ischemic heart disease, even when usual alcohol consumption patterns are light or moderate [4]. Similar outcomes have been observed for ischemic stroke, but less is known about the relationship between binge drinking and other health outcomes, including those which may mediate the relationship between alcohol use and cardiovascular disease risk (e.g., hypertension, high cholesterol and obesity).

Most research on alcohol and health has been conducted in Western countries [4], but concern is now beginning to focus on emerging alcohol consumption in middle income countries such as Thailand [7]. Alcohol consumption in Thailand increased steadily from 1999 to 2008 and by 2008 was among the highest in Southeast Asia [8], although still lower than in most Western countries. Thai alcohol consumption differs from most western countries. Overall, consumption is lower, spirits are popular, and wine consumption is minimal but increasing [8, 9]. The health costs of alcohol consumption in 2006 were estimated to be 2 % of GDP [10]. With the loss of 5.3 % of disability adjusted life years attributed to its consumption, alcohol is now the third most significant health risk for Thai men [11].

The health consequences of increasing drinking trends in Thailand are still incompletely understood. As consumption has increased so has mortality from liver disease as well as morbidity and mortality from traffic accidents [11]. However, little is understood about the impact of alcohol consumption on NCDs in Thailand. As Thailand has transitioned from a traditional subsistence economy to an urban industrial economy, NCDs, including stroke and ischemic heart disease, have increased and now rank in the top 10 causes of Thai mortality [12]. High intensity drinking occasions, or binge drinking are a concerning pattern of alcohol consumption in Thailand [13], but as yet, there has been no investigation of whether this pattern of drinking is related to NCDs [14].

We undertook this study to examine the relationship between alcohol consumption patterns and NCDs in Thailand. Our study focused on a large sample of adult Open University students undertaking distance learning. They are a group of well-educated, aspirational working and middle-class Thais who are leading the way in the transition to a modern lifestyle [15]. They live nationwide embedded in their local communities and are of typical (modest) means, and are capable of reporting complex personal information. We document the relationship between heavy drinking occasions, or binge drinking, and various NCDs in this cohort, as well as documenting other lifestyle characteristics associated with binge drinking (including lifestyle risk factors for NCDs).

## Methods

### Generating the Thai cohort

Thai Cohort Study members were recruited in 2005 from 200,000 Sukhothai Thammathirat Open University students, adult distance learners residing throughout Thailand and remaining in their communities while studying a university course. A 20 page baseline questionnaire was mailed to participants. The questionnaire reached all students who had completed at least their first semester, with 87,151 (at least 44 %) responding. The questionnaire was developed iteratively, tested and modified in Thailand. Details of data processing procedures can be found elsewhere [15]. Missing responses were minimal (between 0.01 % and 3.7 % for the variables used in the current analysis.) Missing responses for alcohol consumption (*n* = 1,716) and those with incongruent answers (*n* = 182) were excluded from analysis. For most analyses there were data for approximately 85,250 cohort members.

On recruitment, the Thai Cohort had a median age of 29 years; 54 % were female, 52 % resided in urban regions, 95 % were Buddhist and the median annual income was US$2,550 [15]. Respondents were similar to other Sukhothai Thammathirat Open University students [16]. Although cohort members are more educated and somewhat younger than the general Thai population they are similar to the adult Thai population in their socio-economic level, their national geo-demographic distribution, and their religion and ethnic mix [15]. We analyzed these baseline data for information about frequency and quantity of alcohol consumption and wide-ranging associations with socio-demographic status, lifestyle and health status.

The study was approved by the Sukhothai Thammathirat Open University Research and Development Institute (protocol 0522/10) and the Australian National University Human Research Ethics Committee (protocol 2004344, 2009/570). Informed written consent was provided by all participants with additional verbal consent obtained from guardians for those aged under 18.

### Measures

#### Alcohol consumption patterns

Two questions on drinking alcohol replicated those used in the *“Thai survey of cigarette smoking and alcohol drinking 2004”* and measure typical use (not available in English). The translated Thai 2004 survey of alcohol consumption asked about glasses which are assumed to be the equivalent of Thai standard drinks.

For our study, alcohol consumption patterns were assessed using the following two questions: 1) “Have you ever drunk alcohol (‘No, never’, ‘Occasional social drinker’, ‘Current regular drinker’, ‘Used to drink before, now stopped’)?”; and, 2) “How many glasses of alcohol do/did you have in one sitting when you are/were drinking (‘less than 2 glasses’, ‘2–3 glasses’, ‘4–5 glasses’, ‘6 glasses or more’^1^)?”.

Participants who answered “No, never” in the first question were “never drinkers” (*n* = 22,527) and those who answered “Used to drink before, now stopped” were “ex-drinkers” (*n* = 7,548). The current drinkers (i.e. remaining participants) were classified based on whether or not they drank 4 or more glasses on a single occasion, because this indicates drinking to intoxication in a Thai person [17] and corresponds to international definitions of binge drinking [6]. This yielded four categories of current drinkers: “occasional light drinkers” (*n* = 26,861) who drank less than 4 glasses per occasion; “regular light drinkers” (*n* = 490) who drank less than 4 glasses per occasion; “occasional heavy drinkers” (*n* = 24,152) who reported drinking 4+ glasses per occasion; “regular heavy drinkers” (*n* = 3,675) who drank 4+ glasses per occasion.

### Disease

Occurrence of NCDs was established by presenting a list of 25 health conditions to respondents and asking “…whether you have ever been told by a doctor that you have this condition *(please tick all that apply)”.* Health conditions included “hypertension”, “high cholesterol” and “liver disease”. Several health conditions associated with alcohol consumption were excluded from the analysis because there were too few cases to analyse with multivariable logistic regression (cancer, cerebrovascular disease and diabetes) [18]. Self-reported hypertension has been investigated in the cohort and found to be of high sensitivity and good overall accuracy [19].

Body mass index (BMI) was calculated using self-reported height and weight [weight(kg)/height (m)^2^] and categorized using suggested Asian cut-points for obesity (overweight 23 to < 25, obese ≥ 25) [20]. Validity of self-reported height and weight has been confirmed in a group of STOU students who were comparable to the cohort [21].

### Demographics and lifestyle factors

The questionnaire included demographic variables (age, sex, area of residence, income, prior education, working status, marital status and religion), and lifestyle variables (smoking, food preference and physical activity). Urbanization (‘countryside’ vs. ‘city/town’) was assessed based on the participant’s current residence and urban migration was based on change from the participant’s residence at age 10–12 years (urban-urban, urban-country, country-urban, country-country). Food preferences included average serves per day of vegetables and fruit and typical frequency of eating food/desserts with coconut milk (< monthly, 1–3 times per month, 1–2 times per week, 3 or more times per week) and deep fried food (< monthly, 1–3 times per month, 1–2 times per week, 3 or more times per week). Physical activity measures included the typical number of strenuous exercise sessions per week (defined as more than 20 min of exercise with rapid heartbeat, e.g. heavy lifting, digging, aerobics or fast bicycling, running, soccer, takraw), and time spent sitting per day. The usual number of hours of sleep was counted.

### Statistical analysis

All analyses were performed with SAS 9.3 software. The distribution of drinking patterns was cross tabulated with socio-demographic and behavioral variables with trends for proportions and means evaluated by the Cochran-Armitage and Kruskal Wallis tests, respectively. Ex-drinkers were not included for the trend test, because the Cochran-Armitage trend test is suitable for tables with ordered columns indicating the dosage level. The prevalence of each NCD by drinking patterns was examined using the chi-square test. For each NCD, multivariate logistic regression models were used to calculate odds ratios (ORs) and 95 % confidence intervals (CIs) for each drinking category relative to never drinkers. Model 1 adjusted for age and sex, model 2 adjusted for age, sex and demographic variables and model 3 adjusted for age, sex, demographic variables and lifestyle variables. (Table 3 shows results for the 3 models). All tests were two-sided and significance was set at p < 0.05.

## Results

### Alcohol consumption in Thai cohort members

Overall 65 % of the cohort were occasional or regular drinkers. This included 78 % of males and 53 % of females. Twenty six percent of the cohort had never drunk alcohol, this being much more common for women (43 %) than men (13 %) while 9 % were ex-drinkers.

The distribution of alcohol consumption patterns among the participants is shown in Table 1. Larger quantities of alcohol consumed was strongly associated with tobacco use: current smoking was reported by 1 % of never drinkers, 4 % of occasional light drinkers, 19 % of occasional heavy drinkers and 39 % of regular heavy drinkers. Urban migration was also associated with heavier alcohol consumption. Higher levels of alcohol consumption were seen in the North region. Lower alcohol consumption was related to living in the south of Thailand, being Muslim and having a low income. For men, greater alcohol consumption was associated with working in the public sector and being married, whereas drinking by women was more common among younger unmarried women who were living with their partner and who were living in Bangkok.

**Table 1:** Distribution of individual attributes by alcohol consumption categories (*n* = 85,253)

	Never drinkers (*n* = 22,527)	Occasional light drinkers (*n* = 26,861)	Occasional heavy drinkers (*n* = 24,152)	Regular heavy drinkers (*n* = 3,675)	p for trend	Ex-drinkers (*n* = 7,548)
Age(yrs): mean (SD)	29.3 (8.3)	30.2 (8.1)	30.7 (7.7)	33.7 (7.9)	<.0001	32.3 (9.2)
Sex						
Men	18	32	74	94	<.0001	57
Women	82	68	26	6	<.0001	43
Education level, %						
<=High school	48	45	51	52	<.0001	55
Diploma/certificate	26	29	28	26	0.07	22
> = University	26	26	22	22	<.0001	23
Income(Baht), %						
<=7000	53	42	34	21	<.0001	45
7001-20000	38	48	54	63	<.0001	42
>20000	9	10	10	16	<.0001	13
Marital status, %						
Single	62	56	49	36	<.0001	50
Living with partner	3	5	7	7	<.0001	6
Married	35	39	44	57	<.0001	44
Region, %						
Bangkok	18	18	16	15	<.0001	17
Central/East	33	31	29	29	<.0001	29
North	13	18	23	23	<.0001	19
Northeast	17	21	23	22	<.0001	24
South	20	12	9	10	<.0001	10
Urban migration, %						
Country-Country	47	43	43	39	<.0001	42
Country-Urban	27	33	34	34	<.0001	31
Urban-Urban	22	19	19	22	<.0001	21
Public sector worker, %	44	46	53	59	<.0001	45
Job type, %						
Professional/Manager	18	18	17	19	0.007	18
Skilled/Manual worker	20	21	23	23	<.0001	22
Office assistant	42	42	20	36	<.0001	36
Religion						
Buddhist	87	97	98	98	<.0001	94
Islam	10	1	0.8	0.4	<.0001	3
Smoking, %						
Never smoker	97	85	50	18	<.0001	53
Current smoker	1	4	19	39	<.0001	12
Ex-smoker	1	9	28	38	<.0001	32
Coconut milk, %						
< monthly	14	13	13	17	0.10	15
1–3 times/month	32	34	33	34	0.40	33
1–2 times/week	31	31	31	27	0.07	28
3 or more times/week	23	22	24	22	0.02	25
Deep fried food, %						
<3 times/month	18	17	14	11	<.0001	19
1–2 times/week	31	32	30	26	<.0001	30
3–6 times/week	35	38	41	44	<.0001	35
Daily	16	14	15	19	0.15	16
Vegetable, mean serves/day (SD)	2.0 (1.7)	1.9 (1.5)	1.9 (1.6)	1.8 (1.5)	<.0001	1.9 (1.6)
Fruit, mean serves/day (SD)	3.1 (2.5)	2.8 (2.4)	2.6 (2.4)	2.0 (2.0)	<.0001	2.7 (2.5)
Strenuous exercise, mean times/week (SD)	1.6 (3.2)	1.7 (3.3)	2.2 (3.3)	2.1 (3.1)	<.0001	1.9 (3.2)
Hours sleeping/day, mean (SD)	6.9 (2.3)	6.9 (2.3)	6.9 (2.2)	7.0 (2.2)	0.007	6.9 (2.3)
Watching TV, mean hours/day (SD)	3.0 (1.9)	2.9 (1.8)	3.0 (1.9)	3.0 (1.9)	<.0001	2.8 (1.9)
Sitting, mean hours/day (SD)	6.5 (3.9)	6.6 (3.9)	6.4 (3.8)	6.2 (3.7)	<.0001	6.7 (3.9)

Light drinkers consumed < 4 glasses/occasion; heavy drinkers consumed 4+ glasses per occasion. Occasional vs. regular drinking reflected self-report of being an “occasional social drinker” vs. “current regular” drinker respectively. Numbers given are column percentages. Percentages for job status, religion, smoking and urban migration do not add to 100 % due to the other choices (not shown) in these categories

Consuming 4+ glasses per occasion (i.e., occasional heavy drinkers and regular heavy drinkers) showed a small but significant association with other health risks for NCDs, including poor diet, with lower fruit and vegetable intake and more frequent consumption of deep fried food (Table 1). The positive association between higher alcohol consumption and more exercise/lower sitting time was mainly found amongst men. Analyzing this association separately for men and women showed a small but significant relationship between greater alcohol consumption and sedentary behavior (more time sitting, watching television and less time doing strenuous exercise; analyses not shown) for both sexes.

### Alcohol patterns and disease

NCDs affected up to one-fifth of participants included in this analysis and were more common among men than women (Table 2). The prevalence of high cholesterol, high blood pressure, liver disease and obesity increased with greater alcohol consumption among men. For women the same pattern was noted for NCDs, although data for women drinkers were sparse.

**Table 2:** Number of cases and prevalence (%) of non-communicable disease and injury by alcohol consumption patterns^a^

NCD outcome x Sex		Drinking category^c^					*p*-value	Total
		Never drinker	Occasional light drinkers	Occasional heavy drinkers	Regular heavy drinkers	Ex-drinkers		
Men								
High cholesterol	n (%)	308 (8)	956 (11)	2,099 (12)	610 (18)	560(13)	<.0001	4,605 (12)
High blood pressure	n (%)	220 (5)	515 (6)	1,158 (6)	336 (10)	387 (9)	<.0001	2,671 (7)
Obesity^b^	n (%)	787 (19)	1,692 (20)	4,059(23)	1,041 (30)	999 (24)	<.0001	8,689 (22)
Liver disease	n (%)	127 (3)	347 (4)	951 (5)	298 (9)	351 (8)	<.0001	2,105 (5)
Women								
High cholesterol	n (%)	1,134 (6)	1,145 (6)	307 (5)	21 (9)	227 (7)	<.0001	2,841 (6)
High blood pressure	n (%)	467 (3)	461 (3)	165 (3)	10 (4)	104 (3)	0.162	1,209 (3)
Obesity^b^	n (%)	1,876 (10)	1,653 (9)	664 (11)	21(9)	375 (12)	<.0001	4,596 (10)
Liver disease	n (%)	281 (2)	381 (2)	177 (3)	8 (3)	105 (3)	<.0001	953 (2)

^a^Light drinkers consumed < 4 glasses/occasion; heavy drinkers consumed 4+ glasses per occasion. Occasional vs. regular drinking reflected self-report of being an “occasional social drinker” vs. “current regular” drinker respectively

^b^BMI ≥ 25Kg/m^2^

^c^Sample size of alcohol categories by sex: Men (*n* = 38,700), never drinkers 4,077, occasional light drinkers 8,630, occasional heavy drinkers 17,840, and regular heavy drinkers 3,441; Women (n = 46,550), never drinkers 18,453, occasional light drinkers 18,229, occasional heavy drinkers 6,311, and regular heavy drinkers 234

As shown in Table 3, associations between alcohol consumption patterns and NCDs were analyzed using 3 progressive models of confounder adjustment. After adjustment for age and sex (model 1), both occasional heavy drinkers and regular heavy drinkers had greater odds of all NCDs compared to never drinkers. These relationships persisted after adjustment for other demographic factors (model 2). The final model further adjusted for lifestyle factors (model 3). In model 3, occasional heavy drinkers had a small increase in the risk of hypertension, and high cholesterol (OR ≈ 1.2), while regular heavy drinkers had a slightly larger increase in the risks of these non-communicable diseases (OR ≈ 1.5). The odds ratios for liver disease were elevated with all three patterns of alcohol consumption, from 1.2 for occasional light drinkers to 1.5 for occasional heavy drinkers and 2.0 for regular heavy drinkers (Table 3).

**Table 3:** Multivariate logistic regression models^a^ showing Odds Ratio (OR) linking alcohol category^b^ (relative to never drinkers) to NCD outcomes

Occasional light drinkers	Model 1			Model 2			Model 3		
	OR	95 % CI	*P* value	OR	95 % CI	*P* value	OR	95%CI	*P* value
High cholesterol	1.13	(1.05–1.22)	0.002	1.07	(0.97–1.16)	0.11	1.06	(0.96–1.16)	0.18
High blood pressure	1.00	(0.90–1.11)	0.99	1.01	(0.90–1.14)	0.84	1.02	(0.90–1.15)	0.82
Obesity	0.89	(0.84–0.94)	<.0001	0.93	(0.87–0.99)	0.03	0.92	(0.86–0.98)	0.01
Liver disease	1.32	(1.16–1.49)	<.0001	1.25	(1.09–1.44)	0.002	1.23	(1.06–1.42)	0.006
Occasional heavy drinkers	Model 1			Model 2			Model 3		
	OR	95 % CI	*P* value	OR	95 % CI	*P* value	OR	95%CI	*P* value
High cholesterol	1.38	(1.27–1.49)	<.0001	1.21	(1.11–1.33)	<.0001	1.19	(1.08–1.32)	0.0005
High blood pressure	1.23	(1.10–1.37)	0.0002	1.22	(1.08–1.38)	0.0002	1.18	(1.03–1.35)	0.02
Obesity	1.16	(1.10–1.23)	<.0001	1.16	(1.08–1.24)	<.0001	1.08	(0.99–1.16)	0.06
Liver disease	1.80	(1.59–2.04)	<.0001	1.58	(1.37–1.83)	<.0001	1.46	(1.25–1.71)	<.0001
Regular heavy drinkers	Model 1			Model 2			Model 3		
	OR	95 % CI	*P* value	OR	95 % CI	*P* value	OR	95%CI	*P* value
High cholesterol	1.92	(1.70–2.15)	<.0001	1.57	(1.38–1.79)	<.0001	1.50	(1.31–1.73)	<.0001
High blood pressure	1.63	(1.41–1.89)	<.0001	1.66	(1.41–1.95)	<.0001	1.54	(1.29–1.85)	<.0001
Obesity	1.48	(1.35–1.62)	<.0001	1.46	(1.32–1.61)	<.0001	1.26	(1.13–1.41)	<.0001
Liver disease	2.76	(2.34–3.25)	<.0001	2.36	(1.96–2.83)	<.0001	2.03	(1.66–2.48)	<.0001
Ex–drinkers	Model 1			Model 2			Model 3		
	OR	95 % CI	*P* value	OR	95 % CI	*P* value	OR	95%CI	*P* value
High cholesterol	1.10	(0.99–1.22)	0.07	1.18	(1.06–1.33)	0.004	1.15	(1.02–1.30)	0.02
High blood pressure	1.29	(1.13–1.46)	0.0001	1.34	(1.16–1.56)	<.0001	1.24	(1,06–1.46)	0.009
Obesity	1.03	(0.95–1.11)	0.53	1.11	(1.01–1.21)	0.02	1.05	(0.95–1.15)	0.37
Liver disease	2.39	(2.08–2.76)	<.0001	2.34	(1.99–2.75)	<.0001	2.13	(1.79–2.53)	<.0001

^a^Model 1: Adjusted sex and age Model 2: Further adjusted for education, income, occupation, marital status, urban-rural residence, region and religion

Model 3: Further adjusted for lifestyle factors (smoking, food/dessert with coconut milk, deep fried food, vegetable and fruit intake, frequency of strenuous exercise, hours sleeping, hours watching TV hours, hours sitting). ^b^Drinking categories (see Methods) are shown in footnote for Table 2

## Discussion

In this large sample of transitional Thais, who are leading the way to modern lifestyles nationwide, we found higher prevalence alcohol consumption than among the general Thai population, with 78 % of men and 53 % of women reporting current drinking (cf. corresponding figures of 48 % and 13 % for the 2007 Thai National Household Survey for Substance and Alcohol Use [13]). This suggests that although average alcohol consumption is lower in Thailand than in Western countries [22], drinking alcohol may have become relatively more common among this aspirational working and middle-class section of the adult Thai population.

Within this cohort, both occasional and regular heavy drinking (4+ glasses on a single occasion) were associated with increased odds of important non-communicable diseases (high cholesterol, hypertension, liver disease and obesity). Ex-drinkers had higher ORs of liver disease compared to current drinkers in the fully adjusted model [OR 2.13 95 % CI 1.79–2.15]. This result may reflect reverse causation if people stopped drinking after being diagnosed with liver disease. Because this is a cross-sectional analysis, it is not possible to infer a causal relationship between drinking and NCDs, although we were able to show that this association persisted after statistical adjustment for a wide-range of demographic factors and lifestyle risk factors for NCDs (including exercise and eating habits). Consistent with previous research, drinking alcohol was strongly associated with tobacco smoking [14]. We also found that drinking was associated with sedentary behaviour, poor diet, and urban migration, all of which are symptomatic of a health transition to an affluent urbanised lifestyle, which corresponds with the leading causes of mortality in Thailand now being due to chronic disease [15].

These findings suggest that consumption of four or more glasses of alcohol, even occasionally, may be an important modifiable risk factor for NCDs in Thailand. This pattern of binge drinking has been previously recognized as a potentially risky in Thailand [13], particularly among younger Thai males [14]. In this study, we have shown that it is, in addition, a potential contributor to the increasing prevalence of NCDs in Thailand. Elevated rates of dyslipidemia, hypertension and obesity among occasional and regular heavy drinkers could increase the rate of mortality from coronary heart disease or cerebrovascular disease, which are leading causes of death in Thailand [23].

The burden of alcohol-related disease is growing in non-western middle income countries and Thailand is a good example of the dynamics and trends that drive the accompanying health-risk transition. Consistent with previous research, we found that alcohol intake was associated with greater BMI in heavy drinkers [24, 25], whereas BMI remained low among light drinkers [25, 26]. Heavy drinkers are likely to be more obese because alcohol has a high energy content of 7.1 kcal/g, but has little nutritional value. Our findings are also consistent with previous research showing a dose-response effect of alcohol consumption with hypertension [27–29]. These previous studies have found that the increase in blood pressure is related to the amount of pure alcohol intake, regardless of the type of alcohol beverage consumed [30, 31] or genetic factors governing alcohol metabolism [32, 33]. Other research has found that “binge drinking” (2.2 g/kg in controlled circumstances) increases ambulatory blood pressure [34], risk of cardiovascular mortality [35], HDL-cholesterol and triglyceride levels [36]. However, research on the effects of binge drinking on NCD risk is still limited and our study suggests that even occasional binge drinking of 4 or more glasses per occasion may increase the risk of these NCDs.

Health promotion strategies could target consumption of 4+ glasses per occasion which was found among Thai men particularly those from the North and Northeast regions of the country. Drinking in this region is characterized by domestically produced spirits (including the local spirit Lao Khao) and beer, and is an entrenched component of the local lifestyle [37]. Although social norms around drinking in this region are likely to be a barrier to reducing alcohol consumption; negative attitudes held about drunken behaviour may be a helpful in preventing alcohol-related harms [37]. The alcohol industry, domestic and international, may push the other way [38].

Drinking was less common among Thai women, reflecting cultural standards. It could also reflect women’s reduced capacity to metabolise alcohol, especially low hepatic activity of alcohol dehydrogenase and aldehyde dehydrogenase [39], and low gastric alcohol dehydrogenase activities [40]. Preventative strategies could aim to maintain this low-drinking status, avoiding the poor health outcomes in Western cultures [41]. Women in the study who drank occasionally and heavily (4+ glasses/session) generally belonged to a younger generation who had adopted modern urbanized lifestyles. They tended to be unmarried but living with their partner, middle income earners living in Bangkok and those who had adopted other modern health behaviors (e.g., smoking, unhealthy diets). These women were of child-bearing age, indicating a need to publicize existing guidelines on drinking during pregnancy [9]. The up-take of drinking among younger Thai women mirrors Western cultures, where greater drinking among women reflects their greater economic participation and more tolerant social norms around drinking among women [41, 42].

### Strengths and limitations

A limitation of the study is that it relies on self-reported health outcomes and self-reported consumption of alcohol. The alcohol consumption items we used replicate questions from the “*Thai survey of cigarette smoking and alcohol drinking 2004”*^*1*^. This survey used a Thai word that directly translates into ‘glasses’ in English rather than other types of containers such as bottles, or "drinks". Consequently, we can estimate and compare respondents’ alcohol consumption but we are unable to determine their absolute alcohol intake. From our measures we derived categories for drinking patterns that relate to health outcomes in other studies [4].

Face to face interviews with picture prompts enable quantification of alcohol consumption, but our study was too large and our participants too dispersed for such interviews. Over the years, different standard drinks and measurement approaches have been used to obtain self-reported alcohol consumption making comparisons across studies and countries difficult [43, 44].

We aimed to investigate links between drinking and NCDs with special interest in binge-drinking. We were able to rank respondents for intake frequency, but could not make precise measures of volume. While our self-reported categories are imperfect, they do identify heavy drinkers and our outcome data support that. Also we found cultural norms around regular or frequent drinking and noted those who are at risk. Furthermore, our current analytic categories of “occasional” and “regular” drinkers are compatible with those according to the Alcohol Use Disorders Identification Test (AUDIT), a widely used, internationally validated tool of the World Health Organization. Indeed, two AUDIT categories combined (“monthly or less” and “2 to 4 times a month”) are commensurate with our category of “occasional” drinking [45].

The time frame is important when analyzing relationships between alcohol consumption and NCDs. Drinking behaviour is changeable during the life course and it takes time to develop NCDs. Our cross-sectional study focused on alcohol consumption patterns in 2005 and the detectable relationships between these drinking patterns and some NCDs. Causal linkage of alcohol intake and NCDs requires longitudinal study and has been discussed above (see Alcohol patterns and disease).

A particular advantage of this study is that the Thai Cohort Study participants are distance learning students, a sophisticated group adept at responding to postal questionnaires. Self-report in the Thai Cohort Study has been validated for several measures including height and weight, and hypertension [19, 21]. Due to the small number of women in our study in the heavy drinking category, we have not stratified our results by gender, although significant gender differences are discussed.

Although the Thai Cohort Study is not chosen to directly represent the Thai population, being younger and better educated, it taps into a group of Thais who are community embedded, and generally reflective of the Thai population in terms of other social and economic characteristics [15, 16]. Health transitions seen within this cohort of educational achievers indicate the future direction of the Thai population. Alcohol consumption was higher than found in the Thai National Alcohol Survey. Aside from our sample reflecting a more aspirational group of Thais who may be likely to have higher levels of alcohol consumption than the general population, several differences between the methods used in the Thai cohort study and the national survey may account for this discrepancy. The national survey assessed alcohol use in the past 30 days, and it included adolescents below the legal age for drinking (12–65 years), both of which would have resulted in lower prevalence estimates. The face-to-face interviews used in the national survey may have also elicited under estimates of alcohol consumption due to embarrassment about heavy drinking in the Thai culture, especially among women.

## Conclusions

We conclude that both occasional and regular heavy alcohol consumption is associated with the growing burden of non-communicable disease in Thailand. There is an opportunity to prevent the burden that alcohol has placed on many Western cultures [46]. While men are most at risk, it may also be useful to target Thai women who have not yet taken up harmful patterns of drinking with health promotion campaigns. In Thailand, alcohol prevention initiatives could reinforce cultural norms which protect people from harmful alcohol consumption. The Thai experience provides an important lesson for other middle income countries transitioning toward more affluent, often more Western, ways of life.

## Footnotes

1 The word ‘glasses’ is used throughout this paper to mean glasses of alcohol

## References

[1] Lim SS, Vos T, Flaxman AD, Danaei G, Shibuya K, Adair-Rohani H (2012). A comparative risk assessment of burden of disease and injury attributable to 67 risk factors and risk factor clusters in 21 regions, 1990–2010: a systematic analysis for the Global Burden of Disease Study 2010. The Lancet.

[2] Rehm J, Room R, Graham K, Monteiro M, Gmel G, Sempos CT (2003). The relationship of average volume of alcohol consumption and patterns of drinking to burden of disease: an overview. Addiction.

[3] Rehm J, Taylor B (2006). Alcohol consumption, stroke and public health--an overlooked relation?. Addiction.

[4] Rehm J, Baliunas D, Borges GL, Graham K, Irving H, Kehoe T (2010). The relation between different dimensions of alcohol consumption and burden of disease: an overview. Addiction.

[5] Roerecke M, Rehm J (2012). The cardioprotective association of average alcohol consumption and ischaemic heart disease: a systematic review and meta-analysis. Addiction.

[6] Courtney KE, Polich J (2009). Binge Drinking in Young Adults: Data, Definitions, and Determinants. Psychol Bull.

[7] Murray CJ, Vos T, Lozano R, Naghavi M, Flaxman AD, Michaud C (2012). Disability-adjusted life years (DALYs) for 291 diseases and injuries in 21 regions, 1990-2010: a systematic analysis for the Global Burden of Disease Study 2010. [Erratum appears in Lancet. 2013 Feb 23;381(9867):628 Note: AlMazroa, Mohammad A, Memish, Ziad A]. Lancet.

[8] Global Health Observatory Data Repository. Levels of Consumption: Recorded alcohol per capita consumption, from 1990. Data by country [http://apps.who.int/gho/data/node.main.A1025?lang=en&showonly=GISAH]

[9] Center for Alcohol Studies. เครื่องดื่มแอลกอฮอล์ และผลกระทบ ในประเทศไทย ปี 2556(Alcohol consumption situation and impact on Thailand 2013). Thai Ministry of Public Health: Bangkok, 2013. pp17-19

[10] Thavornccharoensap M, Teerawattanonanon Y, Ythasamut J, Lertpitakpong C, Thitiboonsuwant K, Neramitpitagkul P (2010). The economic costs of alcohol consusmption in Thailand, 2006. BMC Public Health.

[11] Thamarangsi T (2006). Thailand. Alcohol today. Addiction.

[12] Porapakkham Y, Rao C, Pattaraarchachai J, Polprasert W, Vos T, Adair T (2010). Estimated causes of death in Thailand, 2005: implications for health policy. Population Health Metrics.

[13] Assanangkornchai S, Sam-Angsri N, Rerngpongpan S, Lertnakorn A (2010). Patterns of alcohol consumption in the Thai population: Results of the National Household Survey of 2007. Alcohol Alcohol.

[14] Assanangkornchai S, Saunders JB, Conigrave KM (2000). Patterns of drinking in Thai men. Alcohol Alcohol.

[15] Sleigh AC, Lim L, Kjellstrom T, McMichael AJ, Dixon J, Banwell C (2008). Cohort profile: The Thai cohort of 87 134 Open University students. Int J Epidemiol.

[16] Seubsman S, Yiengprugsawan V, Sleigh A, and the Thai Cohort Study Team (2012). A Large National Thai Cohort Study of the Health-Risk Transition based on Sukhothai Thammathirat Open University Students. ASEAN J Open Distance Learn.

[17] Lekskulchai V, Rattanawibool S (2007). Blood alcohol concentrations after “one standard drink” in Thai healthy volunteers. J Med Assoc Thai.

[18] Peduzzi P, Concato J, Kemper E, Holford TR, Feinstein AR (1996). A simulation study of the number of events per variable in logistic regression analysis. J Clin Epidemiol.

[19] Thawornchaisit P, De Looze F, Reid CM, Seubsman S, Sleigh A, Thai Cohort ST (2013). Validity of self-reported hypertension: findings from the thai cohort study compared to physician telephone interview. Global J Health Sci.

[20] Low S, Chin MC, Ma S, Heng D, Deurenberg-Yap M (2009). Rationale for Redefining Obesity in Asians. Ann Acad Med Singapore.

[21] Lim LLY, Seubsman S, Sleigh A (2009). Validity of self-reported weight, height, and body mass index among university students in Thailand: implications for population studies of obesity in developing countries. Popul Health Metrics.

[22] 2014 National Survey on Drug Use and Health ( NSDUH) State Estimates of Substance Use and Mental Disorders [http://www.samhsa.gov/disorders/substance-use].

[23] Boutayeb A, Boutayeb S (2005). The burden of non communicable diseases in developing countries. Int J Equity Health.

[24] Lourenço S, Oliveira A, Lopes C. The effect of current and lifetime alcohol consumption on overall and central obesity. In*.*, vol. 66. England: Nature Publishing Group; 2012: 813-818. 10.1038/ejcn.2012.20 22378229

[25] Sayon-Orea C, Martinez-Gonzalez M, Bes-Rastrollo M (2011). Alcohol consumption and body weight: a systematic review. Nutr Rev.

[26] Arif AA, Rohrer JE (2005). Patterns of alcohol drinking and its association with obesity: data from the third national health and nutrition examination survey, 1988–1994. BMC Public Health.

[27] Corrao G, Bagnardi V, Zambon A, La Vecchia C (2004). A meta-analysis of alcohol consumption and the risk of 15 diseases. Prev Med.

[28] Nakanishi N, Yoshida H, Nakamura K, Suzuki K, Tatara K. Alcohol consumption and risk for hypertension in middle-aged Japanese men. In*.*, vol. 19; 2001: 851-855. 10.1097/00004872-200105000-00003 11393666

[29] Tsuruta M, Adachi H, Hirai Y, Fujiura Y, Imaizumi T (2000). Association between alcohol intake and development of hypertension in Japanese normotensive men: 12-year follow-up study. Am J Hypertens.

[30] Okamura T, Tanaka T, Kadowaki T, Ueshima H, Yoshita K, Chiba N (2004). Specific alcoholic beverage and blood pressure in a middle-aged Japanese population: The High-Risk and Population Strategy for Occupational Health Promotion (HIPOP-OHP) Study. J Hum Hypertens.

[31] Zilkens RR, Burke V, Hodgson JM, Barden A, Beilin LJ, Puddey IB (2005). Red wine and beer elevate blood pressure in normotensive men. Hypertension.

[32] Puddey IB, Beilin LJ (2006). Alcohol is Bad for Blood Pressure. Clin Exp Pharmacol Physiol.

[33] Yamada Y, Sun F, Tsuritani I, Honda R (2002). Genetic differences in ethanol metabolizing enzymes and blood pressure in Japanese alcohol consumers. J Hum Hypertens.

[34] Seppä K, Sillanaukee P (1999). Binge drinking and ambulatory blood pressure. Hypertension.

[35] Sull JW, Yi SW, Nam CM, Choi K, Ohrr H (2010). Binge drinking and hypertension on cardiovascular disease mortality in Korean men and women: a Kangwha cohort study. Stroke (00392499).

[36] Rimm EB, Fosher K, Stampfer MJ, Criqui M, Williams P (1999). Moderate alcohol intake and lower risk of coronary heart disease: Meta-analysis of effects on lipids and haemostatic factors. Br Med J.

[37] Moolasart JCS (2012). Drinking culture in the Thai-Isaan context of northeast Thailand Southeast Asian. J Trop Med Public Health.

[38] O’Brien P (2013). Australia’s double standard on Thailand’s alcohol warning labels. Drug Alcohol Rev.

[39] Chrostek L, Jelski W, Szmitkowski M, Puchalski Z (2003). Gender-related differences in hepatic activity of alcohol dehydrogenase isoenzymes and aldehyde dehydrogenase in humans. J Clin Lab Anal.

[40] Frezza M, Di Padova C, Pozzato G, Terpin M, Baraona E, Lieber CS (1990). High blood alcohol levels in women: the role of decreased gastric alcohol dehydrogenase activity and first-pass metabolism. N Engl J Med.

[41] Keyes KM, Li G, Hasin DS (2011). Birth cohort effects and gender differences in alcohol epidemiology: a review and synthesis. Alcohol: Clin Exp Res.

[42] Keyes KM, Schulenberg JE, O’Malley PM, Johnston LD, Bachman JG, Li G (2012). Birth cohort effects on adolescent alcohol use: The influence of social norms from 1976 to 2007. Arch Gen Psychiatry.

[43] Kerr WC, Stockwell T (2012). Understanding standard drinks and drinking guidelines. Drug Alcohol Rev.

[44] Dufour MC (1999). What is moderate drinking. Alcohol Res Health.

[45] Babor TF, Higgins-Biddle JC, Saunders JB, Monteiro MG (2001). The Alcohol Use Disorders Identification Test (AUDIT).

[46] Rehm J, Mathers C, Popova S, Thavorncharoensap M, Teerawattananon Y, Patra J (2009). Global burden of disease and injury and economic cost attributable to alcohol use and alcohol-use disorders. Lancet.

